# Supplementary material for: A decentralized approach for the aerial manipulator robust trajectory tracking
Source: PLoS One. 2024 Mar 7;19(3):e0299223. doi: 10.1371/journal.pone.0299223 (PMC10919597; doi:10.1371/journal.pone.0299223)
Supplement: Attachment — Submitted filename: Response to Reviewers.pdf [file pone.0299223.s001.pdf]

Reviewers' comments with authors' replies for the paper :

"A decentralized approach for the aerial manipulator robust trajectory tracking."

(Ref. No. PONE-D-23-34692)

Y. E. Tlatelpa-Osorio, H. Rodríguez-Cortés and J. Á. Acosta

January 12, 2024

We want to thank the associate editor and the reviewers for the opportunity to revise and resubmit our paper. We reproduce the associate editor and reviewers' comments in bold font and immediately reply point-by-point in the sequel. The changes on the new version of the paper are also highlighted. All the cited works refer to the References section of this letter.

Reply to Rewiever #1

This paper aims to propose a new control strategy for an unmanned aerial manipulator (UAM). First, an equivalent model is proposed to equate the 2-revolute links robotic manipulator model to the 1-revolute, 1-prismatic links robotic manipulator model. Then based on this equivalent model, this paper designs a distributed controller for UAM for tracking.

Comment. Thanks for the time you devoted to revise our work.

After careful reading, I have the following concerns.

1. The background introduction of the article is very detailed, but it seems that it is not clearly stated in the contribution section of the article which problems existing in the above-mentioned existing work are solved by this paper.

Response. Thanks for this comment, which made us realize we needed to clarify this contribution in the manuscript. We have rewritten part of the introduction section to highlight the problem with previous works and our contribution.

We notice that in most works addressing the Aerial Manipulator control by the decentralized approach, the interaction between the two dynamic sub-systems, robot arm, and UAV, is not studied, just neglected or somehow compensated (see, e.g. [1], [2], [3], [4], [5] and references therein), either it is only assumed that the dynamic interaction between the manipulator and the aerial vehicle can be treated as exogenous disturbances acting on each sub-system or neglected. Thus, as far as we know, the only work theoretically treating the interaction is in [6], but only at the kinematic level of the robot arm, which means that, in practice, the analysis is only valid for slow movements when the accelerations can be neglected.

This interaction is essential as the UAV dynamics are a part of the robotic arm dynamics, and the Newton-Euler Recursive algorithm helped us clarify it. Thus, in our proposed contribution, we deeply analyze the interaction at a dynamic level, not just at the robot arm kinematics, as in [6]. This study has two main new outcomes: 1) being able to demonstrate that the interaction between the two subsystems can be treated as exogenous perturbations by proposing a novel equivalent transformation that allows designing an estimator for the torques and forces of the interaction, and 2) the estimator paves the way to design a controller capable of tracking prescribed trajectories with exponential convergence, a robust property highly desirable in practice. We emphasize that this analysis is missing in the literature, normally avoided with assumptions such as slow motion, which are not practical because there may be external disturbances such as gusts of wind that cause high accelerations. Indeed, in the simulation section, we show good performance under unexpected external wind gusts, thanks to the in-the-loop estimator.

2. The structure of the article is quite messy, and I didn't find any basis for such an equivalent transformation. I also found no description of how this equivalence method simplifies the stability analysis.

Response. Thanks for your comments. The content is reorganized, and some of the section's names have been modified as follows.

Section: UAM dynamic model. Subsection: Quadrotor dynamic model. Subsection: Robotic manipulator dynamic model.

Section: Decentralized robust control strategy. Subsection: Quadrotor exogeneous disturbances estimator. Subsection: Quadrotor position and attitude control. Subsection:Robot arm controller based on the equivalent model. Subsection: UAM closed loop dynamics. Subsection: Stability analysis.

Section: Numerical simulations.

In the beginning of Section Decentralized robust control strategy, we have added the following paragraph:

The control design is divided into two control loops: the inner loop is a state feedback controller that customizes the robotic manipulator effects on the aerial vehicle as an exogenous disturbance (C4D), generating a decentralized UAM dynamic model. In contrast, the outer control loop uses the decentralized model and independently applies control strategies for the

quadrotor and robotic arm. We first present the C4D state feedback and then the estimator of the quadrotor exogeneous moments and forces (EQEMF).

We have re-state a phrase to better clarify the difference between the two closed-loop systems, from: The UAM closed-loop dynamics (10)–(12) with the controller (13)–(15) results into: *The UAM dynamics (10)–(12) with the inner loop controller RC4D (13)–(15) results in ...*

In regards of the comment about the stability analysis simplification we can say that the proposed coordinate transformation reveals the exogenous nature of the interaction forces in the equivalent dynamics, and therefore enables the design of the estimator and the subsequent stability analysis.

3. In the section describing the UAM dynamics model, it is written that this paper considers a robotic manipulator to have two revolute joints, but in the simulation section, it is stated that the proposed controller is designed considering a quadrotor with a manipulator composed of a revolute joint and a prismatic joint.

Response. We thank this reviewer for this comment; after re-reading it, we agree that the explanation is unclear. One of the main contributions of this work is that we re-formulate the two revolute joints manipulator (1R2R) by a prismatic and a revolute joints (1R1P). Then, we design the control strategy for the 1R1P manipulator. Therefore, using the diffeomorphism transformation of Definition 1 in the simulations section, we can 'mathematically translate' the designed control strategy on the 1R2R joint manipulator for its implementation. Although this step might seem straightforward, it has been included for clarity of presentation.

4. The description of the simulation scene settings in the simulation part of the paper is insufficient, and the figures of the simulation scenes do not show what the author wants to prove.

Response. Thanks for the comments, now we added more information about the simulation scenario as well as an improvement on the figures presented.

5. The writing format of this paper is very irregular, especially the references section.

Response. Thank you. We have carried out a complete revision of the manuscript to improve the reading.

Reply to Reviewer #2

This article proposes a Decentralized Robust Control Strategy for unmanned

aerial manipulators in the presence of external disturbances. The author proposes an equivalent dynamic model that facilitates analysis and can be used to analyze the complete properties of the system. Overall, this article has sufficient workload and also provides a complete analysis and simulation process.

Comment. Thank you for the time you devoted to reviewing our work and for highlighting the key points and the overall workload assessment.

Here are my suggestions.

1.The paper fail to mention the differences and advantages and disadvantages between centralized and decentralized control, and why decentralized control strategies should be adopted.

Response. Thanks for your comment. In the introduction section, we have pointed out a reference that highlights the characteristics of the centralized and decentralized approaches. There is no crucial reason to follow one method or the other; however, the decentralized approach fits better with the attributes of the proposed disturbance estimator.

2.The format of the paper still needs to be checked and modified. For example, in the last paragraph of the Introduction, the Roman numerals after the section cannot be displayed.

Response. Thanks for this observation, We carried out a complete revision of the manuscript and attended the problems with the format.

3.The images in the paper are very blurry, and I cannot see the parameters of UAV and other values in the last image clearly.

Response. We changed the last image and added two tables, one with the physical parameters and other with the controller gains. Also, the quality of the images containing the error dynamics time evolution were revised.

References

[1] Marco Tognon, Burak Yüksel, Gabriele Buondonno, and Antonio Franchi. Dynamic decentralized control for protocentric aerial manipulators. In *2017 IEEE ICRA*, pages 6375–6380. IEEE, 2017.

[2] Alejandro Suarez, Pablo Ramon Soria, Guillermo Heredia, Begoña C Arrue, and Anibal Ollero. Anthropomorphic, compliant and lightweight dual arm system for aerial ma-

nipulation. In *Intelligent Robots and Systems (IROS), 2017 IEEE/RSJ International Conference on*, pages 992–997. IEEE, 2017.

[3] J. Á. Acosta, C. R. de Cos, and A. Ollero. A robust decentralised strategy for multi-task control of unmanned aerial systems. application on underactuated aerial manipulator. In *2016 International Conference on Unmanned Aircraft Systems (ICUAS)*, pages 1075–1084, June 2016.

[4] Mariana Felisa Ballesteros-Escamilla, David Cruz-Ortiz, Isaac Chairez, and Alberto Luviano-Juárez. Adaptive output control of a mobile manipulator hanging from a quadcopter unmanned vehicle. *ISA transactions*, 94:200–217, 2019.

[5] Fabio Ruggiero, Miguel Angel Trujillo, R Cano, H Ascorbe, Antidio Viguria, C Peréz, Vincenzo Lippiello, Aníbal Ollero, and Bruno Siciliano. A multilayer control for multirotor uavs equipped with a servo robot arm. In *2015 IEEE international conference on robotics and automation (ICRA)*, pages 4014–4020. IEEE, 2015.

[6] JÁ Acosta, CR de Cos, and A Ollero. Accurate control of aerial manipulators outdoors. a reliable and self-coordinated nonlinear approach. *Aerospace Science and Technology*, 99:105731, 2020.
